# Transition Pathway and Its Free-Energy Profile: A Protocol for Protein Folding Simulations

**DOI:** 10.3390/ijms140816058

**Published:** 2013-08-02

**Authors:** In-Ho Lee, Seung-Yeon Kim, Jooyoung Lee

**Affiliations:** 1 Korea Research Institute of Standards and Science, Daejon 305-340, Korea; E-Mail: ihlee@kriss.re.kr; 2 School of Liberal Arts and Sciences, Korea National University of Transportation, Chungju 380-702, Korea; E-Mail: sykimm@ut.ac.kr; 3 Center for In Silico Protein Science, School of Computational Sciences, Korea Institute for Advanced Study, Seoul 130-722, Korea

**Keywords:** molecular dynamics, free energy, reaction coordinate

## Abstract

We propose a protocol that provides a systematic definition of reaction coordinate and related free-energy profile as the function of temperature for the protein-folding simulation. First, using action-derived molecular dynamics (ADMD), we investigate the dynamic folding pathway model of a protein between a fixed extended conformation and a compact conformation. We choose the pathway model to be the reaction coordinate, and the folding and unfolding processes are characterized by the ADMD step index, in contrast to the common *a priori* reaction coordinate as used in conventional studies. Second, we calculate free-energy profile as the function of temperature, by employing the replica-exchange molecular dynamics (REMD) method. The current method provides efficient exploration of conformational space and proper characterization of protein folding/unfolding dynamics from/to an arbitrary extended conformation. We demonstrate that combination of the two simulation methods, ADMD and REMD, provides understanding on molecular conformational changes in proteins. The protocol is tested on a small protein, penta-peptide of met-enkephalin. For the neuropeptide met-enkephalin system, folded, extended, and intermediate sates are well-defined through the free-energy profile over the reaction coordinate. Results are consistent with those in the literature.

## 1. Introduction

Understanding the protein folding process inside a living cell is one of the most important issues in modern biology [1,2], and an accurate description of the protein folding pathway has remained a challenging problem in molecular biology for the last few decades. Obtaining both theoretical and experimental information about folding processes as well as transiently populated intermediate states located between folded and unfolded states, requires the development of new theoretical and experimental methods. In recent years, computational approaches based on the energy function of a protein conformation have provided valuable knowledge about the protein folding mechanism along with various experimental techniques such as kinetics study with circular dichroism measurement, NMR spectroscopy, X-ray crystallography, and/or Fersht Φ value analysis [3–5]. The reliability of a folding simulation may depend on the adequacy of the potential energy function used. Therefore, to stay in touch with reality, theoretical calculations should be tested against experimental data as much as possible.

When performing a simulation, although there are issues with the accuracy of potential energy function used, one important factor to overcome is that the simulation time should be long enough compared to the actual reaction time of the phenomenon under investigation. Therefore, developing a computational method that either extends the time scale of the simulation or expedites the reaction itself is rather important. In order to draw reliable and consistent conclusions out of a simulation, the overall results of the simulation, especially on kinetic and thermodynamic properties of a protein, should not depend on the length of the simulation time scale. Thus, through rigorous folding pathway simulations, one can provide meaningful assessment of the relevance of a potential energy function used, and if necessary, modification or fine-tuning of the energy function becomes feasible to describe real proteins more accurately.

Recently, various algorithms and hardware for molecular simulations have been developed and applied to protein folding studies. However, carrying out justifiable molecular simulations of proteins is rather demanding, and such simulations are limited to relatively small proteins. One major bottleneck for computational study is the time-scale gap between simulations and experiments. Shaw’s group has developed a specialized supercomputer, called Anton, which greatly accelerates the execution of atomistic molecular dynamics (MD) simulations [6]. Recently, Lindorff-Larsen *et al.* [7] used state-of-the-art MD simulations to investigate the folding mechanisms of 12 proteins. They found that during barrier crossing, the long-range contacts that establish the protein’s overall fold form early along with the formation of a considerable amount of secondary structures and surface burial. Another difficulty is that, in general, it is difficult to define a good reaction coordinate for description of protein folding/unfolding dynamics from/to an arbitrary extended conformation [8].

In this work, we take two separate algorithms for the investigation of the protein folding mechanism and thermodynamic analysis without much fine-tuning. Recently, we have developed and tested an algorithm that provides a protein folding pathway model with the boundary-value formulation of Newtonian dynamics [9–13]. The first algorithm we use is the action-derived molecular dynamics (ADMD) method [9,10], and it is used to generate protein folding pathways. Low energy-barrier transition pathways are generated by carrying out ADMD simulations. The advantage of ADMD lies in the fact that it does not require the selection the reaction coordinate in advance. ADMD trajectories are generated by optimizing an action with a specific choice of the target energy. Generated atomic trajectories are shown to be rather close to the Verlet trajectory for the whole trajectory even with a relatively large time step.

The second algorithm we use is the replica-exchange molecular dynamics (REMD) method [14,15], which is used for the thermodynamic analysis of the pathway. Using the pathway obtained by ADMD simulations, we estimate the free-energy profile along the pathway. The conformations obtained from the ADMD simulation provide a set of diverse conformations for the subsequent REMD simulation. Then, we use the REMD method to quantify the thermodynamic properties of protein, *i.e.*, free-energy profile and transition states along the pathway.

In this work, we propose a methodology to construct free-energy profile along the folding pathway of a protein by combining ADMD and REMD methods. The approach is tested to simulate the folding process of a small peptide met-enkephalin. In the following sections, first we introduce ADMD, REMD, and related descriptions on details. Next, we discuss the folding mechanism of met-enkephalin obtained by the combined simulations of ADMD and REMD. The results are compared with existing theories and experiments. A summary is given in the conclusion section.

## 2. Results and Discussion

Before analyzing details of ADMD simulation results, the limitation of ADMD approach should be discussed. The goal of ADMD is to obtain the most probable pathway connecting two end structures. Here the choice of end structures can be problematic, especially since the unfolded state cannot be fully represented by a single structure. Here we used an extended structure as the representative of the unfolded state, but a rigorous treatment should consider multiple unfolded structures. Likewise, obtaining the most probably pathway is not sufficient enough to understand the general folding phenomenon, especially for studying small peptides. However, even with these limitations, proper application of ADMD can provide an insight on the folding mechanism as discussed in this section below.

Here we describe the folding pathway of met-enkephalin generated by combining ADMD with REMD. First, we describe the folding pathway of met-enkephalin investigated by ADMD. Second, we investigate the free-energy profile along the folding pathway generated by ADMD. The free energy profile of a folding process can strongly depend on the choice of the reaction coordinate. By choosing the series of conformational changes obtained by ADMD simulation as the reaction coordinate, we obtain the free-energy profile along the ADMD trajectory.

Met-enkephalin (Tyr-Gly-Gly-Phe-Met) is a small neurotransmitter peptide with morphine-like activity and it is found in the central nervous system. Met-enkephalin has been the object of many computational studies of identifying its low-energy conformations [16–20]. NMR spectroscopy experiments on the peptide in dimethyl sulfoxide support the presence of a turn at Gly^3^ and Phe^4^ with a hydrogen bond between Gly^2^ and Met^5^ [21–24]. A recent NMR experiment suggests that the solution structure of met-enkephalin constitutes an ensemble of diverse structures including extended conformations with large fluctuations [25]. No unique native structure has been determined for met-enkephalin in infrared and fluorescence experiments [26,27]. Similar conclusions are obtained by circular dichroism and absorption study of met-enkephalin in solution [28,29]. On the other hand, an extended conformation is obtained by X-ray crystallography [30,31], which is probably due to the crystal packing effect of the peptide.

Identification of the minimum-energy conformation of met-enkephalin has been an issue in the literature, especially in computational studies. However, finite temperature effects on the conformational variation of the small peptide should be properly considered. The flexibility of the peptide is also related to the fact that it binds to at least two receptors of the central nervous system. In addition, met-enkephalin’s conformation is medium-dependent. Thus, a study on the multiple stable structures of the peptide and dynamic connectivity between these structures can be a biologically important subject. In our previous study, folding dynamics of met-enkephalin is investigated by ADMD method [32]. We used the same potential and ADMD parameters as in [32]. Here, we have performed additional new ADMD simulations. No data from [32] are used in the analysis of present work. Using only two input structures of the initial (extended) and the final (folded) conformations, dynamic pathways between the two given conformations have been successfully obtained. In the current work, by studying the free-energy profile along the ADMD folding pathway, we find that there exists an ensemble of populated structures along the pathway.

### 2.1. ADMD Simulations: Pathway Analysis and Reaction Coordinate

**Figure 1:**
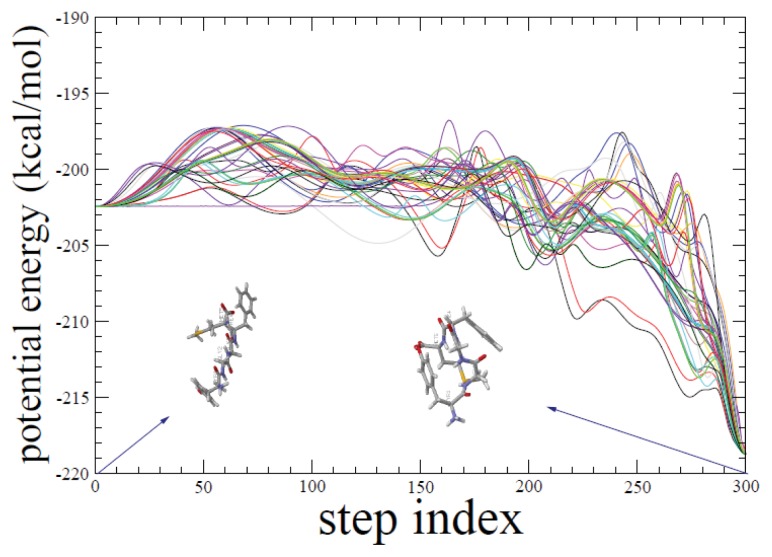
Fifty independent potential-energy variations for met-enkephalin are shown along the action-derived molecular dynamics (ADMD) step index. The same initial and final conformations are used for all ADMD simulations. The potential-energy variations clearly show the existence of multiple pathways. There exist many alternative folding routes that connect the extended and compact peptide conformations. Two conformations used as inputs for ADMD simulations are shown. The initial (final) conformation shown at *j* = 0 (300) is prepared by applying local energy minimization to the fully-extended (compact) structure. We observe that one of the ADMD trajectories is of almost constant potential energy in the first half of the trajectory. In this part, the kinetic energy is rather high. It should be noted that the total energy (sum of kinetic energy and potential energy) is conserved along all ADMD pathways.

**Figure 2:**
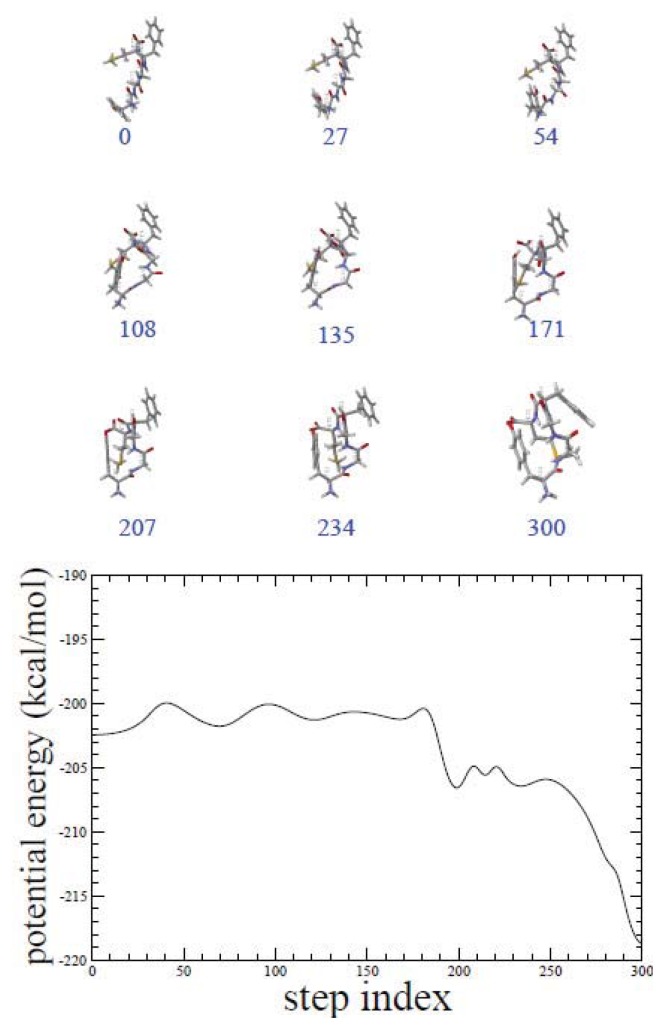
Snapshots of the lowest potential-energy-barrier pathway for met-enkephalin are shown. Potential-energy fluctuation is shown along the ADMD step index. As the index increases, the potential-energies related to the pathway model undergo fluctuation. Potential-energy fluctuations associated with the most-fit (lowest potential-energy barrier profile) pathway from the simulations are shown.

We have performed 50 independent ADMD simulations, each of which produced a relatively low potential-energy pathway with an energy barrier in the range of 2.4–6.0 kcal/mol. Initial pathways for ADMD simulations were prepared in a random fashion to sample as many variations as possible in the pathway. In Figure 1, 50 potential-energy fluctuations are shown along the ADMD step index. The same set of initial and final conformations is used for all ADMD simulations. The potential-energy fluctuations clearly show the existence of multiple folding pathways for met-enkephalin. In Figure 2, we show the lowest energy-barrier pathway out of 50. In the formalism of ADMD, low-energy barrier trajectories serve as probable transition pathways, and we assume that the lowest-potential-energy pathway represents the most relevant pathway. This pathway model is used for a further analysis in the present work.

Since atomic motions in the ADMD pathway model statistically satisfy the microscopic time reversal symmetry imposed on the Verlet trajectory, the current ADMD formulation can describe forward as well as reverse reactions (folding and unfolding events). In the current ADMD simulations, calculated trajectories deviate from the Verlet trajectory with minimal error as discussed in the previous section.

**Figure 3:**
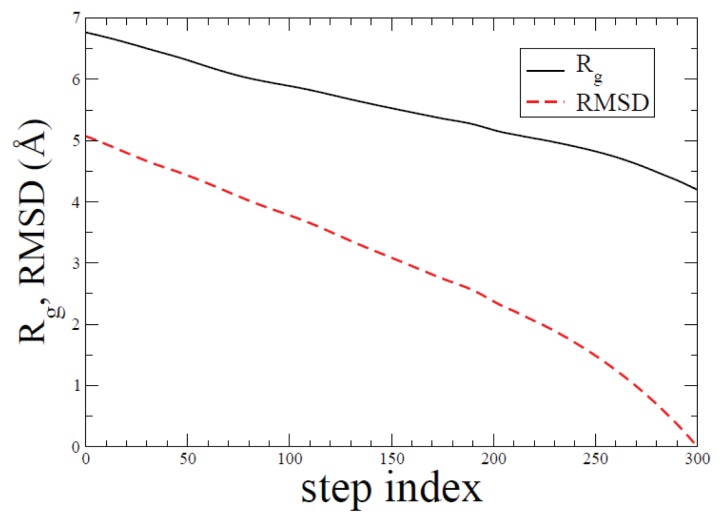
Calculated *R_g_* and root-mean square deviation (RMSD) values in Å are shown along the ADMD step index. We observe that *R_g_* and RMSD are correlated.

A set of characteristic quantities along the folding pathway is shown in Figure 3. Radius of gyration (*R_g_*) and root-mean square deviation from the compact folded conformation are shown. For RMSD calculations, we used all atoms. We have used the quaternion method [33,34] to superimpose two given structures.

**Figure 4:**
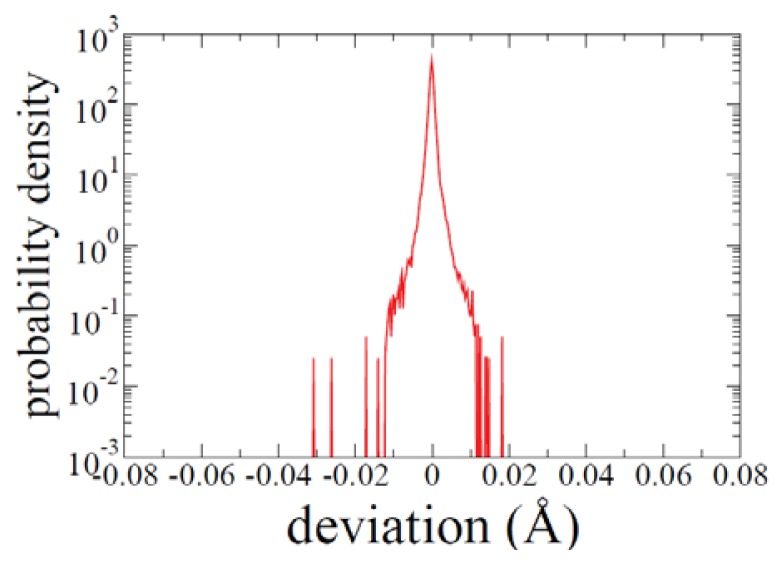
A typical distribution of the scalar “error variables” introduced in the text is shown. Here, for pathway generation, Δ = 8.063 fs, *N* = 75 atoms, and *P* = 300 are used. To obtain the distribution function, 3*N*(*P* − 1) = 67275 scalar “error variables” are used. Calculated distribution of the scalar “error variables” $\{{\vec{ɛ}}_{I,j}\}=2{\vec{q}}_{I,j}-{\vec{q}}_{I,j-1}-{\vec{q}}_{I,j+1}-\frac{\Delta^{2}}{m_{I}}\frac{\partial V(\{{\vec{q}}_{j}\})}{\partial {\vec{q}}_{I,j}}$ is shown. Even with the relatively large time step of Δ = 8.063 fs, the size of the deviation is rather limited.

A typical distribution of the scalar “error variables” introduced in the previous section is shown in Figure 4. An example for measuring the path quality of a transition path connecting two given configurations is shown. Here, for the pathway generation, Δ = 8.063 fs, *N* = 75 atoms, and *P* = 300 are used. Therefore, a total of 3*N*(*P* − 1) = 67275 scalar “error variables” are used to obtain the distribution function.

**Figure 5:**
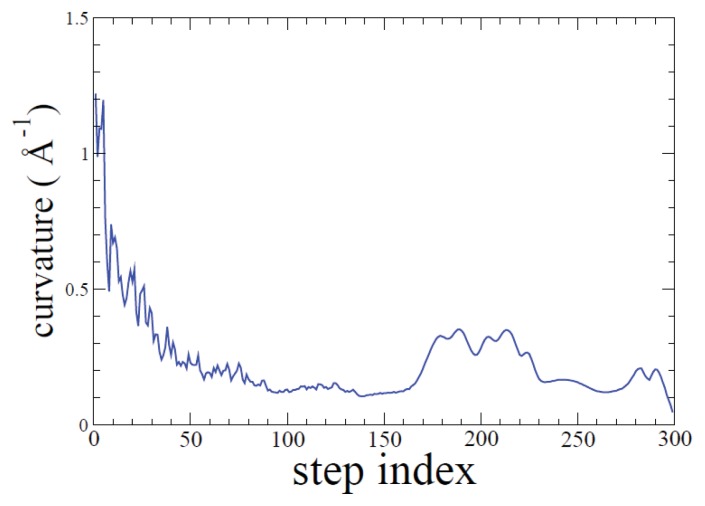
Curvature of the transition pathway calculated by Equation (5) is shown. The radius of curvature ranges between 1.6 and 10.0 Å, and after the step index *j* = 30 the radius is larger than 3 Å. This means that there exist no abrupt changes of atomic motions along the path.

Generated ADMD pathways are smooth. That is, no abrupt changes of the conformation are observed. To demonstrate this, we have calculated the curvature of the transition pathway along the ADMD step index (Figure 5). The curvature is defined as follows.

(1) $$C(j)=\frac{\sqrt{\sum_{I=1}^{N}\left(2{\vec{q}}_{I,j}-{\vec{q}}_{I,j-1}-{\vec{q}}_{I,j+1})\cdot (2{\vec{q}}_{I,j}-{\vec{q}}_{I,j-1}-{\vec{q}}_{I,j+1}\right)}}{\sqrt{\sum_{I=1}^{N}\left({\vec{q}}_{I,j}-{\vec{q}}_{I,j-1})\cdot ({\vec{q}}_{I,j}-{\vec{q}}_{I,j-1}\right)}\sqrt{\sum_{I=1}^{N}\left({\vec{q}}_{I,j}-{\vec{q}}_{I,j+1})\cdot ({\vec{q}}_{I,j}-{\vec{q}}_{I,j+1}\right)}}.$$

The curvature of a straight line is zero, and the curvature of a circle is inversely proportional to its radius. In Figure 5, we find that the curvature of the trajectory stays in the range of 0.1–0.6 Å^−1^. This means that the radius of curvature associated with the pathway is in the range of 1.6–10.0 Å. In summary, the generated ADMD pathway model, presented as a series of similar conformations, does not undergo any abrupt changes.

### 2.2. REMD Simulations: Thermodynamic Analysis

**Figure 6:**
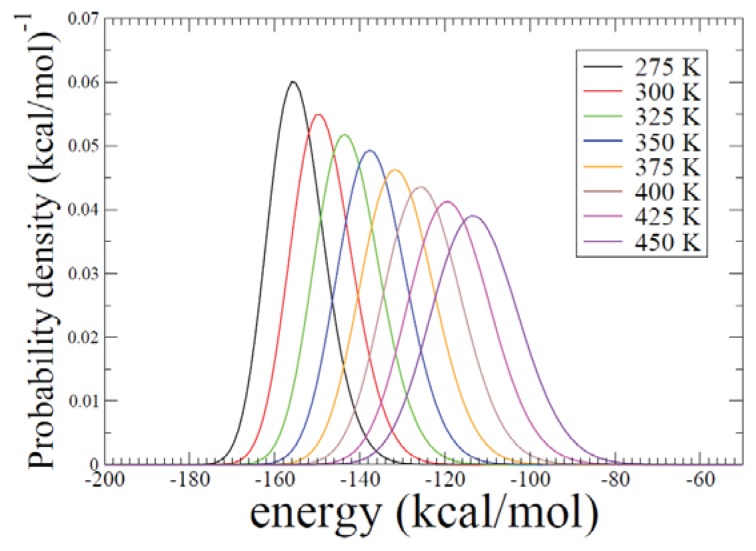
Probability density functions obtained from the REMD simulation are shown for eight temperatures. Significant overlaps are observed between neighboring temperatures.

By using REMD simulations we have obtained probability density functions (PDFs) for a set of temperatures as shown in Figure 6. In the figure, we observe a significant amount of energy overlaps in the coverage of PDF between two adjacent temperatures *T_i_* and *T_i + 1_*.

**Figure 7:**
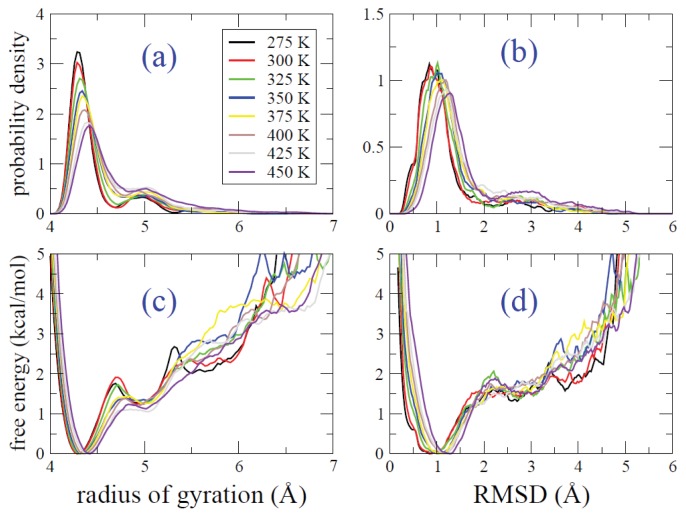
Distribution functions of the radius of gyration (**a**) and RMSD (**b**) obtained from REMD simulation are shown. Free energy functions in terms of the radius of gyration (**c**) and RMSD (**d**) are shown.

Radius of gyration distribution as a function of temperature obtained from REMD simulation is shown in Figure 7a. The average of radius of gyration increases as temperature increases. Similarly, RMSD distribution as a function of temperature obtained from REMD simulation is also shown in Figure 7b. Again, we find that the average RMSD increases as temperature increases, indicating that the population of the extended-like structures increases. Free energy against the radius of gyration and RMSD are shown in Figure 7c,d, respectively.

**Figure 8:**
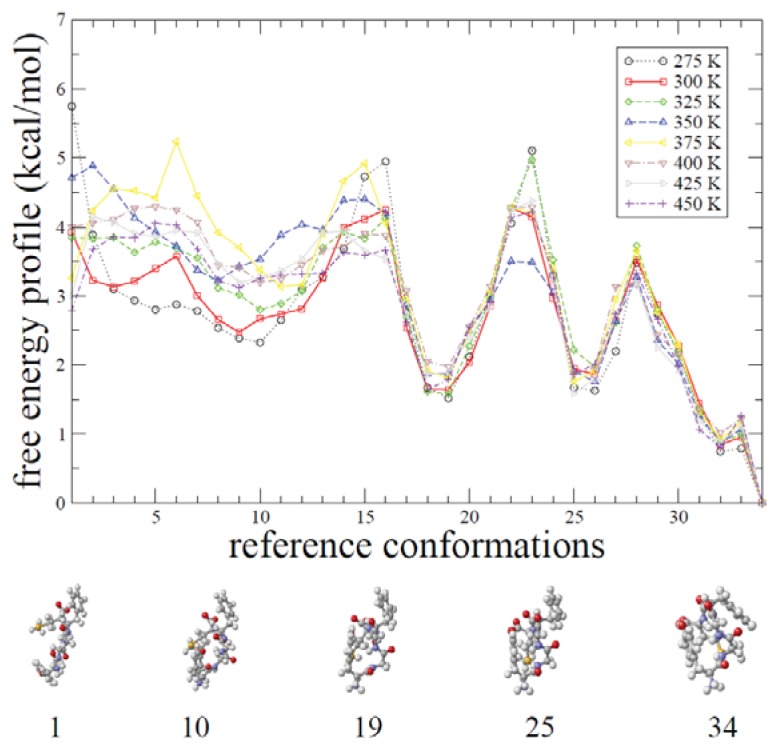
Free-energy profile obtained from REMD simulation based on the 34 “states” (or reference conformations) as described in text are shown. We take the reference energy of the free energy in such a way that its observed minimum is set to zero. Five reference conformations are also shown. The ground state (reference conformation 34) is favored thermodynamically and separated from the other low energy states by free-energy barriers. Reference conformation 1 is the fully extended conformation and marginally stable with a small free energy barrier of about 1 kcal/mol. Three reference conformations 10, 19 and 25 are metastable structures. For the free energy profile convergence test, we have carried out additional 50% of simulation time. The resulting two free energy profiles agree to each other within 1 kcal/mol.

In Figure 8, a free-energy profile projected on the 34 reference conformations is shown. A set of free-energy valleys (reference indices, 1, 10, 19, 25 and 34) can be defined along the profile. These valleys represent peptide conformations with accumulated population during the REMD simulations. These (extended, intermediate, and compact) structures are shown along the free-energy profile over the reaction coordinate. Here, the peptide encounters various quasi-stable intermediate states between extended and compact structures. Non-synchronization between gain in potential energy and loss in conformational entropy results in a multi-basin free energy profile. We demonstrate that the alliance between the two simulation methods, ADMD and REMD, has a great synergistic effect in understanding the protein folding process.

**Figure 9:**
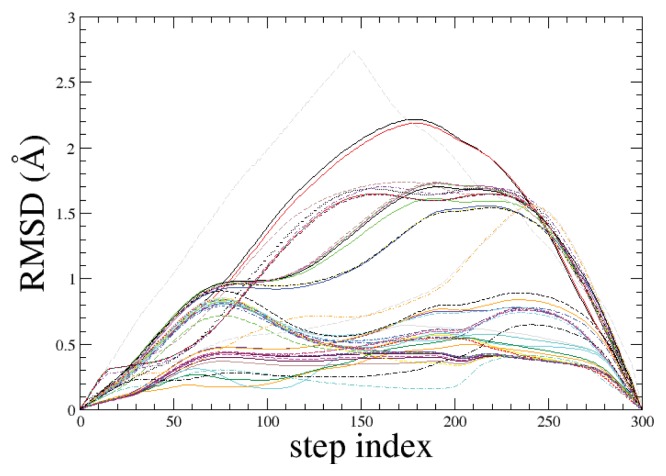
The lowest energy-barrier pathway is compared to the other 49 ADMD pathways by calculating RMSD values between two corresponding conformations. We find that calculated RMSD values are less than 1 Å over all ADMD step indices for 35 pathways out of 49. For the other 14 folding models, calculated RMSD values are below 3 Å.

Although the goal of using ADMD approach is to find the most probable pathway out of many possibilities, we compared the lowest energy-barrier pathway to the other 49 ADMD pathways. We observe significant overlaps between the lowest energy-barrier pathway and the other 49. In order to quantify the overlap between two ADMD pathway models, we calculated a series of RMSD values between two corresponding structures from two folding models. From Figure 9, we find that calculated RMSD values are less than 1 Å over all ADMD step indices for 35 pathways out of 49. For the other 14 folding models, calculated RMSD values are below 3 Å over all step indices. This suggests that although the current ADMD approach may not guarantee to cover all pathways, the most relevant one is likely to be covered, since significant overlap is observed among the majority of 50 ADMD pathways.

One of the key questions remaining is whether the obtained free energy profile can explain populations of diverse conformations found in experiments. The fully extended structure of reference conformation 1 (or 3) is metastable with a small free energy barrier of about 1 kcal/mol as shown in Figure 8. This means that the conformational diversity especially similar to the extended structure is supported by the free energy calculation. In addition, we observe three additional metastable reference conformations, 10, 19 and 25, as intermediate conformations. These conformations represent a series of molecular compaction. Their respective structural stabilities at finite temperature are found in the free energy profile. Possibly as many as four different conformations are locally stable in addition to the final compact structure. This is consistent with NMR experimental results that suggest the existence of an ensemble of flexible structures including extended conformations in the solution structure of the peptide [25].

The relative stability of the locally-stable extended structure fluctuates as the temperature increases. In contrast, the calculated free energy profile demonstrates that, as the temperature increases, there are no dramatic changes in the profile near the compact structure. The barriers between two adjacent free energy basins at temperature 300 K is found to be about 2–3 kcal/mol, indicating that the peptide could be easily populated at two separate basins. The existence of the two well-separated stable conformations in met-enkephalin is consistent with the known affinity of met-enkephalin for distinct opioid receptors.

Experimental studies indicate that met-enkephalin does not take a single compact conformation in the aqueous solution, and an ensemble of various structures is populated [25]. An NMR study supports the presence of a turn at Gly^3^ and Phe^4^ in dimethyl sulfoxide. Kinoshita *et al.* found that a fully extended conformation has the highest stability in water by using the reference interaction site model theory [35]. The reference interaction site model theory is a useful tool to study solvation effects, and when it is combined with replica-exchange Monte Carlo simulations, it is shown that solvent molecules tend to extend the peptide conformation and smooth out its free-energy landscape [36]. Li and Scheraga [37] also concluded that, at room temperature, met-enkephalin in water is likely in an unfolded state. These findings, including the current study, are in qualitative agreement with NMR experimental data [25].

## 3. Theoretical Methods

### 3.1. ADMD Method: Background and Setup Parameters

The proposed protocol is tested on a small protein, penta-peptide met-enkephalin. We used the amber95 all-atom force field with the GB/SA solvent model [38] as implemented in the TINKER package [39]. Two conformations of met-enkephalin, extended and compact, are used as the input for the ADMD simulation. The initial extended conformation was obtained by local optimization of the above energy function starting from the fully extended conformation. Similarly, the final compact conformation is obtained by local optimization of the putative ground-state structure of met-enkephalin reported in the literature [16,40–43]. Here, we note that the initial conformation is an artificial conformation, not a preferred conformation of the peptide.

ADMD simulations are typically used to study rare events such as protein folding by generating the most probably trajectories with fixed initial and final boundary conditions [9,10,44]. Here we describe the essence of the method with some details for self-containedness. In ADMD, a pre-set time interval [0,τ] is divided into *P* slices of equal time length with Δ = τ/*P*. In other words, the time step is set as *t_j_* = *j*Δ with *j* = 0,1,2, …, *P*. In this study we used *P* = 300 and Δ = 8.06 fs. The goal of ADMD is to optimize the objective function in Ref. 10 with 3*N*(*P* – 1) = 67275 degrees of freedom, where the total number of atoms *N* = 75 for met-enkephalin. The atomic mass of each constituent atom (H, C, N, O, and S) is taken from its standard value and the total-energy conservation is taken into account. All atoms are treated as point particles. It should be noted that no artificial constraints on covalent bond lengths and bond angles are employed. The computational task of ADMD is to perform minimization with 3*N*(*P* − 1) independent variables. Potential energy and force calculation is the most time-consuming part of the computation. To avoid various numerical difficulties, a modified action can be used. The modified action is transformed from a functional to a function of *P* − 1 vectors { *q⃗_j_* }, where the index *j* denotes the time frame corresponding to its three-dimensional image. Here, *j* = 0 and *j* = *P* correspond to the given initial and final images, respectively. One can define the discretized Lagrangian of the *j*-th temporal frame as

(2) $$L_{j}=\sum_{I=1}^{N}\frac{m_{I}}{2\Delta^{2}}{\left({\vec{q}}_{I,j}-{\vec{q}}_{I,j+1}\right)}^{2}-V(\{{\vec{q}}_{j}\}).$$

Here, the first term corresponds to the kinetic energy and *V* is the potential energy. *m_I_* is the mass of the *I*-th atom, and *q⃗_I j_*_,_ is the position vector of the *I*-th atom at the *j*-th time frame. Finally, the modified action used in this ADMD becomes

(3) $$\Phi (\{{\vec{q}}_{j}\},E;T)=\sum_{j=0}^{P-1}L_{j}\left(\left\{{\vec{q}}_{j}\right\}\right)\Delta +\mu_{E}\sum_{j=0}^{P-1}{\left(E_{j}-E\right)}^{2}+\mu_{K}\sum_{I=1}^{N}{\left(\langle K_{1}\rangle -\frac{3k_{B}T}{2}\right)}^{2},$$

where <*K_I_*> is the average kinetic energy of the *I*-th atom along the trajectory [9]. *E* is the pre-set target energy of the system. A fictitious temperature *T* controls the kinetic energy of the system, *μ_E_* and *μ_K_* are arbitrarily large constants, and *k_B_* is the Boltzmann constant.

The quality of a generated path can be assessed by calculating its Onsager-Machlup (OM) action [45] defined as follows;

(4) $$S_{OM}=\frac{1}{3N(P-1)}\sum_{I=1}^{N}\sum_{j=1}^{P-1}{\left[2{\vec{q}}_{I,j}-{\vec{q}}_{I,j-1}-{\vec{q}}_{I,j+1}-\frac{\Delta^{2}}{m_{I}}\frac{\partial V(\{{\vec{q}}_{j}\})}{\partial {\vec{q}}_{I,j}}\right]}^{2}.$$

The term inside brackets is zero for a trajectory generated by Verlet algorithm [46]. Therefore, the OM action becomes zero for a true Verlet path in the limit of Δ→0, and it can be used to estimate the proximity of a generated discrete trajectory to its ideal Newtonian trajectory, piece by piece.

With the additional *μ_K_* term in Equation (2), Lee *et al.* [10] demonstrated that the quality of trajectories is much improved in terms of the OM action [45] compared to that from the original action. After the final path is obtained by the action optimization process, one can estimate the quality of the path by calculating *S_OM_* as the sum of 3*N* × (*P* − 1) “error variables” in Equation (3). If Δ is small enough, a Verlet trajectory is guaranteed. The histogram of “error variables” of a typical ADMD path is shown in Figure 4. Even with relatively large Δ, deviation from the ideal Verlet trajectory is shown to be minimal. Elber *et al.* [44] also pointed out that the distribution of “error variables” can be used as a useful indicator for assessing trajectories generated by MD simulations with non-negligible Δ. The current ADMD approach is shown to generate high-quality trajectories with significantly small values of *S_OM_* in the study of protein folding pathways [11–13,32,47] as well as systems in various condensed matter physics [48–56].

### 3.2. REMD Simulations: Setup Details

For an ideal system where the ‘exact’ force field is provided, ADMD paths represent the most probable folding trajectories at folding condition. Generally, obtaining the most probable pathway to a folded state constitutes only half of the folding story, and the other half should be concerned with thermodynamics of folding. Following the philosophy of the ADMD methodology that, when studying a rare event such as protein folding, investigation of the most probably pathway by solving a boundary value problem is a better strategy than unguaranteed attempts for direct observation of the event from conventional MD simulations, we have designed MD simulations to study the thermodynamics of the folding along the pathway generated by ADMD. Therefore, the goal of MD simulations in this study is to estimate the free energy profile along the generated ADMD pathway. Here, we propose that this goal can be achieved by projecting all MD trajectories onto generated ADMD frames. This is based on the assumption that the folding contribution from the phase space far from a generated ADMD pathway is neither indispensable nor heavily biased toward a particular ADMD state (out of *P +* 1 = 301 frames).

In practice, rather than dealing with all *P +* 1 = 301 frames, we have selected a total 34 reference conformations for projection from the ADMD simulation (ADMD step indices *j* = 0, 9, 18, …, 270, 279, 288 and 300). Conformations obtained from MD simulations are categorized according to their similarity with the 34 reference conformations as determined by the lowest RMSD value.

Here, we describe details about the method we employed to estimate the free-energy profile along the pathway generated by ADMD. In order to confine MD simulation trajectories in the proximity of a predetermined pathway (*i.e.*, ADMD pathway), we add the following restraint potential.

(5) $$V_{add}(\{{\vec{q}}_{I}\})=\{\begin{array}{rr} 0 & if\;RMSD<2\;Å,\\ \frac{k}{2}\sum_{I=1}^{N}{\left({\vec{q}}_{I}-{\vec{q}}_{I,0}\right)}^{2} & otherwise. \end{array}$$

root-mean square deviation (RMSD) in Equation (4) corresponds to the lowest one out of 34 superimpositions of the current MD conformation against 34 reference conformations. We denote the closest reference conformation as { *q⃗_I_*_,0_} where *I* is the atomic index of *N* = 75 met-enkephalin atoms. If the current MD conformation is similar to one of the 34 preset conformations within the RMSD value of 2 Å, the above restraint term does not affect the dynamics. When it deviates, *i.e.*, RMSD > 2 Å, the restoring harmonic restraint term in Equation (4) is applied. Here, we used *k* = 2.24 kcal/mol/Å^2^. The penalty function is designed to keep the molecule within the region close to the ADMD transition pathway. We measured the portion of population affected by nonzero V_add_ from Equation (4). We find that only 1.2% of the total population was governed by nonzero V_add_. In addition, we find that there are no specific trajectories that systematically tend to exit the transition tube. This means that neighboring reference conformations are sufficiently close to each other and most transitions observed in the REMD simulation are transitions between them. Almost all populations (98.8%) are sampled within the transition tube (defined by 2 Å range from the pathway) in the present REMD simulation. It should be noted that this penalty term does not affect the relative population along the pathway, since it is zero along the pathway within 2 Å.

The reaction coordinate chosen in this study is along the folding pathway characterized by the ADMD step index. Two successive conformations in the pathway model are quite similar to each other with a small RMSD value in the range of 0.2–0.33 Å. By means of structural clustering, we are able to determine the ensembles of the folding step including the transition state. The connectivity between successive conformations basically represents the folding process of the molecule in an overall view. Within this procedure one can calculate the population of the conformations on the reaction coordinate as the function of temperature considering multiple temperature MD simulations. This should be contrasted to *a priori* reaction coordinates typically used in conventional molecular simulations.

We have considered 8 × 4 MD simulations, initially uniformly sampled from the pathway model, over eight temperatures (275, 300, 325, 350, 375, 400, 425 and 450 K). Then, based on the Metropolis criterion we attempt exchanging two conformations at neighboring temperatures [14,15]. The idea of this method is to make conformations at high temperatures available to the simulations at low temperatures and vice versa. Each MD simulation covered 60 ns while exchange attempts are tried after each MD simulation of 0.1 ns. The exchange is carried out according the conventional probability of

(6) $$P(exchange)=min\left[1,\text{exp}\left\{\left(\frac{1}{k_{B}T_{i}}-\frac{1}{k_{B}T_{j}}\right)\left(V_{i}-V_{j}\right)\right\}\right].$$

Significant overlaps between probability density functions especially at neighboring temperatures are observed in the simulations (see Figure 6). This represents that exchanges between neighboring replicas in the current REMD simulation are properly maintained.

For an integration of the Langevin equations of motion, we used the integration time step of 1 fs. Damping constant of 91 ps^−1^ was used [57] at each temperature used. Multiple MD simulations are prepared by employing the Maxwell-Boltzmann distribution for all temperatures, ranging from *T* = 275–450 K. We used a separate random number seed for each replica and generated random series of atomic velocities. By carrying out a single set of REMD simulations, one can obtain various thermodynamic quantities as the function of temperature [14,15]. It is shown that the REMD method can be successfully applied to the simulation of proteins with complex energy landscape. It should be noted that both ADMD and REMD simulations can be executed in a highly parallel fashion [9,10,14]. To the best of our knowledge, the current work is the first attempt to combine the ADMD method and the REMD method together.

## 4. Conclusions

In this study, we have combined ADMD and REMD to obtain the free-energy profile along the folding pathway. Pathways are generated by solving a boundary-value problem for given initial and final conformations of a molecule in terms of ADMD formalism. By minimizing the action defined by Equation (2), Verlet-like trajectories are obtained. The quality of ADMD-generated pathways, assessed by the Onsager-Machlup action, is shown to be quite satisfactory. Furthermore, atomic motions in the pathway statistically satisfy the microscopic time reversal symmetry imposed on the Verlet trajectory. Consequently, the ADMD formulation has no limitations in describing forward as well as reverse reactions.

We choose the pathway model to be the reaction coordinate along which kinetic behavior is presumed to be described, and so the process of conformational change is characterized by the ADMD step index. In practice, we define a series of conformations, containing sequential information about the conformational change, as the reaction coordinate. This should be contrasted to the common choice of an *a priori* reaction coordinate as often used in conventional studies of molecular conformational changes.

With a generated pathway model by ADMD, the free-energy profile along the folding pathway is generated by applying restrained REMD simulations. Temperature variation of the free-energy profile over the reaction coordinate is readily evaluated by the REMD method. With the reaction coordinate of the conformational change and its free-energy profile, the thermodynamics of conformational variation are described.

The proposed protocol is applied to a small protein, penta-peptide met-enkephalin. We show that the transition pathway of met-enkephalin between extended and compact conformations can be successfully explored by the method. Overall results are consistent with those in the literature.

It should be noted that both ADMD and REMD simulations can be executed in a highly parallel fashion. We believe that the current method can serve as a useful tool for efficient exploration of conformational space and proper characterization of protein folding dynamics. Study of conformational transitions of large and complex systems is now underway.
